# Effects of Increased Water Salinity and Temperature on the Development of Rainbow Trout Fry Syndrome (RTFS) Caused by *Flavobacterium psychrophilum*

**DOI:** 10.1111/jfd.70037

**Published:** 2025-08-14

**Authors:** Valentina L. Donati, Niels Lorenzen, Lone Madsen

**Affiliations:** ^1^ Section for Fish and Shellfish Diseases, National Institute of Aquatic Resources Technical University of Denmark Kongens Lyngby Denmark

**Keywords:** *Flavobacterium psychrophilum*, rainbow trout fry, RTFS, salt water, temperature

## Abstract

*Flavobacterium psychrophilum* , the etiological agent of Rainbow Trout Fry Syndrome (RTFS) and Bacterial Coldwater Disease (BCWD), causes varying degrees of disease and mortality in salmonid aquaculture worldwide. Because its treatment relies on the use of antimicrobials, alternative interventions are of environmental and economic interest. As for other bacterial diseases, environmental factors like water parameters play a crucial role in the development of RTFS. In this study, we investigated the effect of either increased water salinity [1% (10 g L^−1^)] or water temperature (18°C ± 1°C) on the development of RTFS following challenge with *F. psychrophilum* in rainbow trout fry under experimental conditions (either bath challenge or co‐habitation challenge). When implemented after bath challenge, the salinity treatment delayed the appearance of clinical disease, while the temperature treatment reduced the incubation time to appearance of clinical disease. Given the positive effect of increased salinity, we further assessed the effect of the salinity treatment on disease transmission when the fish were exposed to co‐habitation challenge. While intraperitoneally injected seeder fish died within 2 weeks independent of salinity, cohabitant fish kept in water with 1% salinity had a significant increase in survival (42.6%) compared to the positive controls (17.9%). Infected dead and moribund fish were confirmed positive for *F. psychrophilum* . Increasing water salinity thus delayed and partly prevented RTFS. In applied terms, such a delay may give time to achieve antimicrobial susceptibility test (AST) results and initiate treatment before reaching heavy mortalities in a fish batch. Further studies should evaluate the robustness of the preventive effect of this approach, its effect on the microbial communities (fish and farm environment) and whether it could be combined with other measures (e.g., phage therapy). The effect of the warm water was surprising, as *F. psychrophilum* infections are reported at colder temperatures.

## 1 Introduction

The Gram‐negative bacterium *Flavobacterium psychrophilum* (Borg 1948; Bernardet et al. 1996), etiological agent of Rainbow Trout Fry Syndrome (RTFS) and Bacterial Coldwater Disease (BCWD), can cause varying degrees of disease and mortality in salmonid aquaculture worldwide (reviewed by Nematollahi et al. 2003). In Denmark, the first disease case caused by this bacterium is dated 1985 whereas *F. psychrophilum* was for the first time isolated from sick rainbow trout fry in the summer of 1990 (Dalsgaard and Hørlyck 1990; Lorenzen 1991; Lorenzen et al. 1991), and subsequently identified as the etiological agent of RTFS in the period 1991–1994 (Lorenzen and Olesen 1996a). One of the most affected fish species is rainbow trout (*Oncorhynchus mykiss*, Walbaum), the main farmed fish species in Denmark (Ministry of Environment and Food of Denmark and Danish Fisheries Agency 2022). Mortality caused by this bacterial infection ranges from 30%–90% in 0.2–2 g fry (clinical symptoms: pale gills and liver, grey kidney, whitened intestine and enlarged spleen) to 10%–15% in 2–10 g fingerlings (clinical symptoms: enlarged spleen, anaemia, bleeding of the internal organs and of the mouth region). In larger fish (50–200 g), it can develop into a chronic condition with clinical symptoms such as wounds along the body, pale gills and, in some cases, blindness together with a shortened or deformed spine and abnormal swimming behaviour (Lorenzen and Olesen 1996b).

The use of antimicrobial agents has been the treatment of choice for this bacterial infection since its discovery (Bruun et al. 2000, 2003; Schmidt et al. 2000; Dalsgaard et al. 2009). In Denmark, florfenicol has been used since 1996, but resistance to this antimicrobial has been recently detected (Donati and Madsen 2023). To reduce the use of the traditionally used antibiotics and to counteract the spread of antimicrobial resistance, researchers have focused on finding sustainable alternatives to prevent and control the spread of this disease. Some studies have focused on water recirculation and good management procedures (Madsen and Dalsgaard 2008), vaccine development (Long et al. 2013; Sudheesh and Cain 2016; Hoare et al. 2017, 2019; Ma et al. 2019) and phage therapy (Madsen et al. 2013; Christiansen et al. 2014; Donati et al. 2021). Others have assessed the impact of modifying water parameters, such as salinity, pH and water temperature, in reducing/limiting the infection (reviewed by Nematollahi et al. 2003). In Denmark, salt and increased temperature have been suggested as possible options to reduce the bacterial load in fish at different life stages (Jensen et al. 2003). However, fish challenge experiments are needed to evaluate the feasibility and effectiveness of these methods.

With this study, we wanted to investigate the effect of salt water (1%, 10 g L^−1^) and increased water temperature (18°C) in the treatment of RTFS with the further possible application in the field.

## 2 Materials and Methods

### 2.1 Bacterial Isolates

*Flavobacterium psychrophilum* 20‐1298‐14A (serotype Th, ST92, CC‐ST10) was isolated from a Danish rainbow trout freshwater farm in February 2020. The strain originated from the kidney of a rainbow trout, and it was recently characterised (Donati and Madsen 2024 submitted manuscript). The bacteria were stored at −80°C in tryptone yeast extract salts medium (TYES: 0.4% tryptone, 0.04% yeast extract, 0.05% CaCl_2_ × 2H_2_O, 0.05% MgSO_4_ × 7H_2_O (pH 7.2)) (Holt et al. 1993) and glycerol (15%–20%). For in vitro experiments, *F. psychrophilum* 20‐1298‐14A was initially inoculated from the −80°C stock into 25 mL sterile glass tubes containing 5 mL TYES broth (referred as TYES‐B) for 48 h (115 RPM, 15°C) and then 0.5 mL of the culture was further inoculated into either 100 mL of sterile TYES‐B or 100 mL of the tested substrates (250 mL Erlenmeyer flask with cap). For fish experimental infections, *F. psychrophilum* was prepared as described by Madsen and Dalsgaard (1999). Firstly, *F. psychrophilum* 20‐1298‐14A was inoculated from the −80°C bacterial stock into 5 mL TYES‐B placed in 25 mL sterile tubes with lid and incubated at 15°C (115 RPM) for 72 h, after which the cultures were examined microscopically to visually confirm monoculture of *F. psychrophilum*. At this point, 1 mL of the 72‐h old 5 mL‐culture was inoculated into 500 mL sterile Erlenmeyer flask with cap containing 200 mL sterile TYES‐B and incubated for 44–46 h (15°C—115 RPM). Alternatively, 4.25 mL of the 72‐h old 5 mL‐culture was inoculated into 2000 mL sterile Erlenmeyer flask with aluminium foil as cap containing 850 mL sterile TYES‐B for 44–46 h (15°C—115 RPM). According to the volume needed for the bath challenge, an appropriate number of 200 mL/850 mL‐cultures was produced and, when grown (44–46 h), cultures were singularly checked in the microscope and then pooled together in a 2 or 5 L sterile flask with lid prior to colony forming unit (CFU) enumeration and infection procedures. With this method, the bacterial concentration of the 44/46‐h old cultures reaches 10^8^–10^9^ CFU mL^−1^. CFUs were determined by plating serial dilutions of the bacterial suspension on TYES agar (TYES plus 1.1% agar, referred as TYES‐A) in duplicates right before starting the infection challenge procedures. TYES‐A plates were incubated at 15°C for 5 days, and colonies were then counted.

### 2.2 *In Vitro* Experiments: *F. psychrophilum* in Water With 1% Salinity and Increased Temperature

Two independent in vitro experiments to assess the growth/survival of *F. psychrophilum* 20‐1298‐14A with increased water salinity or temperature were set up. Freshwater (FW) and water with a salinity of 1% (10 g L^−1^ sodium chloride) (1%SW) were collected from the fish facilities. Collected water was filter‐sterilised (sterile 0.2 μm cellulose acetate Sartorius Minisart NML Syringe Filters, Fisher Scientific, Denmark) and streaked on blood‐agar and TYES‐A to confirm sterility (no growth was detected after 9 and 13 days of incubation at 20°C on blood‐agar and at 15°C on TYES‐A).

The first in vitro experiment focused on evaluating the survival of *F. psychrophilum* 20‐1298‐14A in 1%SW. Bacteria (0.5 mL of 48 h‐old culture) were inoculated into either 100 mL of sterile TYES‐B or 100 mL of filter‐sterilised 1%SW. Five millilitres of sterile TYES‐B and 1%SW without any *inoculum* were included (25 mL sterile glass tubes). Cultures were incubated at 15°C at 115 RPM, and bacterial growth was followed by CFU enumeration on TYES‐A (2 replicates/time‐point/condition) at 0, 1, 24 and 48 h post inoculation and, finally, at 9 days after inoculation. Optical density (OD) measurements at 520 nm were also performed with a spectrophotometer (UV–VIS Spectrophotometer UV‐1280, Shimadzu Corp.) at the same time points as CFU enumeration (one replicate per condition). The cultures without any *inoculum* were sampled only at the end of the experiment (9 dpi).

In a second in vitro experiment, the growth/survival of *F. psychrophilum* 20‐1298‐14A was evaluated in TYES‐B at 15°C and 20°C and, in 0.2 μm filter‐sterilised FW and 1%SW at 15°C. As for the first experiment, 0.5 mL of a 48 h‐old culture was inoculated in 100 mL of sterile TYES‐B/filter‐sterilised FW/1%SW. After inoculation, the TYES‐B cultures were incubated at 15°C or 20°C (115 RPM) while bacteria inoculated in FW and 1%SW were incubated at 15°C (115 RPM). For each treatment, two duplicate cultures were prepared. Bacterial growth/survival was followed by CFU enumeration on TYES‐A at 0, 21, 45 and 139 h post inoculation. OD measurements at 520 nm were also performed at 0, 18, 21, 24, 42, 45, 48 and 139 h post inoculation for each biological replicate.

### 2.3 Experimental Infection Trials

#### 2.3.1 Fish

Rainbow trout eyed eggs, purchased at a Danish commercial farm (free of notifiable diseases, i.e., bacterial kidney disease (BKD), viral haemorrhagic septicaemia (VHS), infectious pancreatic necrosis (IPN) and infectious haematopoietic necrosis (IHN)), were transported to the Section for Fish and Shellfish Diseases (DTU Aqua, Kgs. Lyngby, Denmark). The Section's facilities comprise a dedicated area for animal experiments, which includes: a unit for the production of experimental pathogen‐free fish with its own recirculation system; a quarantine unit for receiving fish (flow‐through system); a third high‐containment area for conducting infection trials with fish pathogens (flow‐through system) with four separate units, each equipped with separate regulation of water temperature and salinity. Tap water adjusted for hardness is used in the facilities. All wastewater deriving from this third area is pasteurised (https://www.aqua.dtu.dk/english/facilities/fish‐shellfish‐diseases). Water with increased salinity is obtained by dissolving artificial sea salt in freshwater.

Upon arrival, the eyed eggs were disinfected with a 10‐min treatment in an iodine‐based disinfectant and hatched in a pathogen‐free unit. When fish reached the desired size and weight for the experiments, they were transferred to the high‐containment area. Here, fish were randomly divided in 8‐L tanks, each with its own inlet/outlet for water (water temperature of 12°C–13°C, water flow of 2–3 L h^−1^) and air‐supply. The fish were fed twice a day (morning and afternoon) at 2% of the fish weight per day (0.8–1.5 mm feed pellets according to fish size, Inicio Plus, BioMar A/S, Brande, Denmark). Fish were inspected several times a day and aquaria cleaned every day.

After hatching, fish batches were examined for notifiable fish pathogens and bacterial infections in general. For the latter, fish were dissected for bacteriological examinations and swabs from internal organs were streaked both on TYES‐A and blood‐agar. No bacterial growth was detected for the fish batches used in this study.

An overview of the experimental infection trials is presented in Table 1.

**TABLE 1:** Overview of fish experimental infections and treatments tested.

Exp. no.	Treatment tested^a^	Delivery time (dpi)	Rainbow trout			Bacterial challenge	
			Origin	Weight_i_ (g)	Total no. (no. per aquarium)	Method	Dose
1	Salinity (1%)	I. 1 dpi II. At mortality (11)	DK	3–4	541 (60 ± 1)^b^	Bath	2.6 × 10^7^ CFU mL^−1^
	Control	Constant					
2	Salinity (1%)	1 dpi	UK	0.5–0.7	1402 (58 ± 3)^b^	Bath	9.0 × 10^7^ CFU mL^−1^
	Temp. (18°C)	I. 1 dpi II. At mortality (6)					
	Control	Constant					
3	Salinity (1%)	30 days before challenge	UK	1–2	356 (59 ± 2)^b^	Co‐habitation	5.5 × 10^3^ CFU fish^−1^ ^c^
	Control	Constant					

*Note:* Rainbow trout fry were used for the experiments, and bacterial challenge was performed with *Flavobacterium psychrophilum* 20‐1298‐14A. Freshwater was used for control and temperature treatment groups. Negative controls for the infection (named TYES‐B control aquaria) were included in the experiments (see the experimental setup of each experiment for details).

Abbreviations: DK, Denmark; dpi, days post infection; _i_, initial; UK, Northern Ireland (United Kingdom).

^a^ If not indicated, the water temperature was maintained constant at 12°C.

^b^ Average number of fish per aquarium and standard deviation in the parentheses.

^c^ The infection dose was delivered by intraperitoneal injection to 20 out of the 59 ± 2 fish/aquarium.

##### 2.3.1.1 Fish Experiment No. 1 (Danish Eggs)

To investigate the effect of salinity on *F. psychrophilum* infection, three water treatments groups were established in the first experiment:

1. Salinity treatment (1%, 10 g L^−1^ sodium chloride) at 1 dpi [named ST‐1dpi];
2. Salinity treatment (1%, 10 g L^−1^ sodium chloride) at the onset of mortality [named ST‐Mort.];
3. Freshwater [named Ctrl].

In all groups the water temperature was maintained at 12°C for the entire course of the trial. For this experiment, 541 rainbow trout fry (3–4 g) were divided into nine aquaria (60 ± 1 fish/aquarium). Fish in six aquaria were bath challenged with the bacterium (2.6 × 10^7^ CFU mL^−1^, two‐litre volume, 6 h) and the ST treatment was started either the day after the bacterial challenge (ST‐1dpi: 2 aquaria, 1dpi) or at the onset of mortalities (ST‐Mort.: 2 aquaria, 11 dpi). In the other two infected aquaria, the water remained freshwater (Ctrl). Three negative controls for the infection (sterile TYES‐B, two‐litre volume, 6 h) were included where the water was kept as freshwater (1 aquarium) or it was changed to ST (2 aquaria, ST‐1dpi). In the Ctrl group, we decided as earlier to include only one aquarium for the negative infection control as we have never experienced mortality/disease in this kind of group in previous experiments (Donati et al. 2021).

To perform the bacterial bath challenge, the water in the selected aquaria was removed, and fish were left without water for 30 s (stress factor). The stress factor was introduced to enhance susceptibility to infection with the bath challenge method. Bacterial broth (200 mL of 44/46‐h old culture with 2.6 × 10^8^ CFU mL^−1^) was added to 1800 mL of freshwater and fish were left kept in the 2 L bacterial suspension for 6 h (2.6 × 10^7^ CFU mL^−1^, constant air supply, water flow stopped). After the 6‐h bath, aquaria were filled up to 8 L and water flow was then re‐established (2–3 L h^−1^). The following day, around 24 h after the start of the bacterial exposure, aquaria were cleaned and the water exchanged according to daily cleaning procedures. In the ST groups, the inlet water tube of the ST aquaria was moved and connected to the pipes containing water with 1% salinity at the selected time points (1 dpi or at mortality). The water flow was set up to allow a gradual exchange of water (2–3 L h^−1^) and then maintained.

Fish survival was followed over time. Moribund fish were euthanized [overdose of anaesthetic (3‐aminobenzoic acid ethyl ester, MS‐222, 150–200 mg L^−1^ in freshwater, not buffered)] and collected along with dead fish for measurements of weight and length plus bacteriological examination (re‐isolation of the bacterium from brain, kidney and spleen to confirm the infection). The experiment was terminated 47 days post infection (dpi) where fish were euthanized by immersion in an overdose of the anaesthetic and characterised by weight and length measurements plus bacteriological examination [all fish in infected aquaria, only 10 randomly chosen fish in negative control for the infection].

##### 2.3.1.2 Fish Experiment No. 2 (Northern Ireland Eggs)

Fish size and weight can affect the outcome of *F. psychrophilum* infections and once the fish have reached the fingerling size (3–6 g), it may be difficult to obtain clinical disease under experimental conditions (Lorenzen 1994; Madsen and Dalsgaard 1999). Thus, we established a second experiment with smaller rainbow trout fry (0.7 g) to re‐evaluate the effect of water salinity (1%, 10 g L^−1^) including only one time for initiation of the treatment (1 dpi), based on the results of experiment no. 1. Furthermore, we examined the impact of elevating the water temperature to 18°C ± 1°C on development of RTFS when initiated at two different times post infection (at 1 dpi or at onset of mortality).

Overall, four treatments groups were established in this second experiment:

1. Salinity treatment (1%, 10 g L^−1^ sodium chloride) at 1 dpi (12°C) [named ST];
2. Temperature treatment (18°C ± 1°C, freshwater) at 1 dpi [named TT‐1dpi];
3. Temperature treatment (18°C ± 1°C, freshwater) at onset of mortality [named TT‐Mort.];
4. Freshwater (12°C) [named Ctrl].

For this experiment, 1402 rainbow trout fry were divided into 24 aquaria (58 ± 3 fish/aquarium; six aquaria per treatment where three were exposed to the bacterium and the other three not). Fish were first bath challenged (9.0 × 10^7^ CFU mL^−1^ or sterile TYES‐B, 2 L volume, 6 h) following the same procedure used in the first fish experiment. Three negative‐infection controls aquaria (referred as TYES‐B controls) were included for each of the four studied treatments (Ctrl, ST, TT‐1dpi, TT‐Mort.) for a total of 12 TYES‐B control aquaria. Fish survival was followed over time (dead and moribund fish were sampled as in experiment no. 1).

In the ST group, salinity was changed 1 dpi as in experiment no. 1. In the TT groups, the water temperature was risen and maintained by small heaters placed in each aquarium and monitored with the use of loggers (MicroLite USB Temperature data Logger LITE 5032p‐EXT, Fourtec—Fourier Technologies, distributed by Contika, Denmark). In TT‐1dpi group, temperature was gradually risen 1 dpi from 13.01°C ± 0.40°C to 18.44°C ± 0.47°C (TYES‐B controls) and, from 11.85°C ± 0.03°C to 17.72°C ± 0.14°C (infected aquaria) reached in approximately 12 h. Water temperature was then maintained at 18.89°C ± 0.88°C (TYES‐B controls) and 17.68°C ± 0.50°C (infected aquaria) until termination. In the TT‐Mort. group, the temperature was gradually risen at mortality (6 dpi) from 11.09°C ± 0.26°C to 18.01°C ± 0.21°C (TYES‐B controls) and, from 11.91°C ± 0.19°C to 18.34°C ± 0.76°C (infected aquaria), reached in approximately 12 h. Subsequently, the water temperature was maintained at 18.14°C ± 1.42°C (TYES‐B controls) and 18.07°C ± 0.97°C (infected aquaria) until termination.

The experiment was terminated 28 dpi, and fish were euthanized and examined as for experiment no. 1.

##### 2.3.1.3 Fish Experiment No. 3 (Northern Ireland Eggs)

In the last fish experiment, a cohabitation challenge was established to further evaluate the effect of water salinity (1%, 10 g L^−1^) on disease transmission (1.5–2 g fish, 356 fish in total). Because of the very low survival rate observed in the second experiment where fish were challenged with *F. psychrophilum* 20‐1298‐14A by bath, we decided to try with a cohabitation challenge, which is normally very difficult to perform and reproduce with this bacterium. The experiment was set up as a continuation of the previous one and as a small preliminary study. TYES‐B control fish kept in freshwater at 12°C (three aquaria) or in ST at 12°C (three aquaria) from experiment no. 2 were used for this experiment. All six aquaria (three per treatment) were exposed to the bacterium, because a large variation was expected in the survival rate of the fish in the replicates. Because of this and the fact that we normally never observe mortality/disease in our negative controls, we decided not to include negative‐infection controls for this part of the experiment.

In each of the six aquaria, 20 fish were temporarily removed, anaesthetised by immersion (50–80 mg MS‐222 L^−1^ in freshwater, not buffered (Dalsgaard and Bjerregaard 1991)), intraperitoneally injected (IP, 50 μL, 5.5 × 10^3^ CFU fish^−1^) and fin clipped (dorsal caudal fin) to allow recognition. The infection dose was chosen according to the results obtained in the previous median lethal dose (LD_50_) determination of the isolate (Donati and Madsen, submitted manuscript). After being injected, the fish were replaced in their original aquaria. Fish survival was followed over time (dead and moribund fish were sampled as in experiment no. 1). The trial was terminated 48 dpi, and fish were euthanised and examined as for experiment no. 1.

#### 2.3.2 Re‐Isolation of *F. psychrophilum* From Infected Fish

To perform the bacteriological examination of dead/euthanized fish, samples from fish organs (spleen, kidney, brain) were streaked on TYES‐A and incubated at 15°C for 5 days up to 21–28 days. *F. psychrophilum* yellow colonies were then identified. Some randomly chosen yellow colonies were analysed by matrix‐assisted laser desorption/ionisation (MALDI) time of flight (TOF) mass spectrometry (MS) (Bruker) to confirm the identity of *F. psychrophilum* (Jansson et al. 2020).

#### 2.3.3 Ethics Statement

The animal study was reviewed and approved by the Animal Experiments Inspectorate of Denmark (Dyreforsøgstilsynet, permission n. 2019‐15‐0201‐00159).

### 2.4 Statistical Analysis

Kaplan–Meier survival analysis and statistical analysis of clinical signs data were performed using GraphPad Prism version 10.1.1 for Windows, GraphPad Software, San Diego, CA, United States, www.graphpad.com. In the Kaplan–Meier survival analysis, data from singular replicate aquaria were merged since the difference in survival between the replicates was ≤ 20% (Amend 1981; Midtlyng 2016). Comparison of survival curves was performed with the Log‐rank (Mantel–Cox) test and the Gehan‐Breslow‐Wilcoxon test. In case of comparison of more than two survival curves, each individual comparison was performed and its significance evaluated with the Bonferroni‐corrected threshold (0.05/no. of comparison; the curves are significantly different if the *p*‐value is lower than the Bonferroni‐corrected threshold). Statistical significance of observed clinical signs among different treatment groups were analysed. Normality was at first assessed with the Shapiro–Wilk test. Comparisons between two or more groups were performed with un‐paired *t*‐test or ANOVA (normal distribution) or Kruskal–Wallis (non‐normal distribution). *p*‐values (*p*) below 0.05 were considered significant. In case of multiple comparisons, *p*‐values were adjusted for Dunnett or Dunn's corrections for data with a normal and non‐normal distribution, respectively.

## 3 Results

**FIGURE 1:**
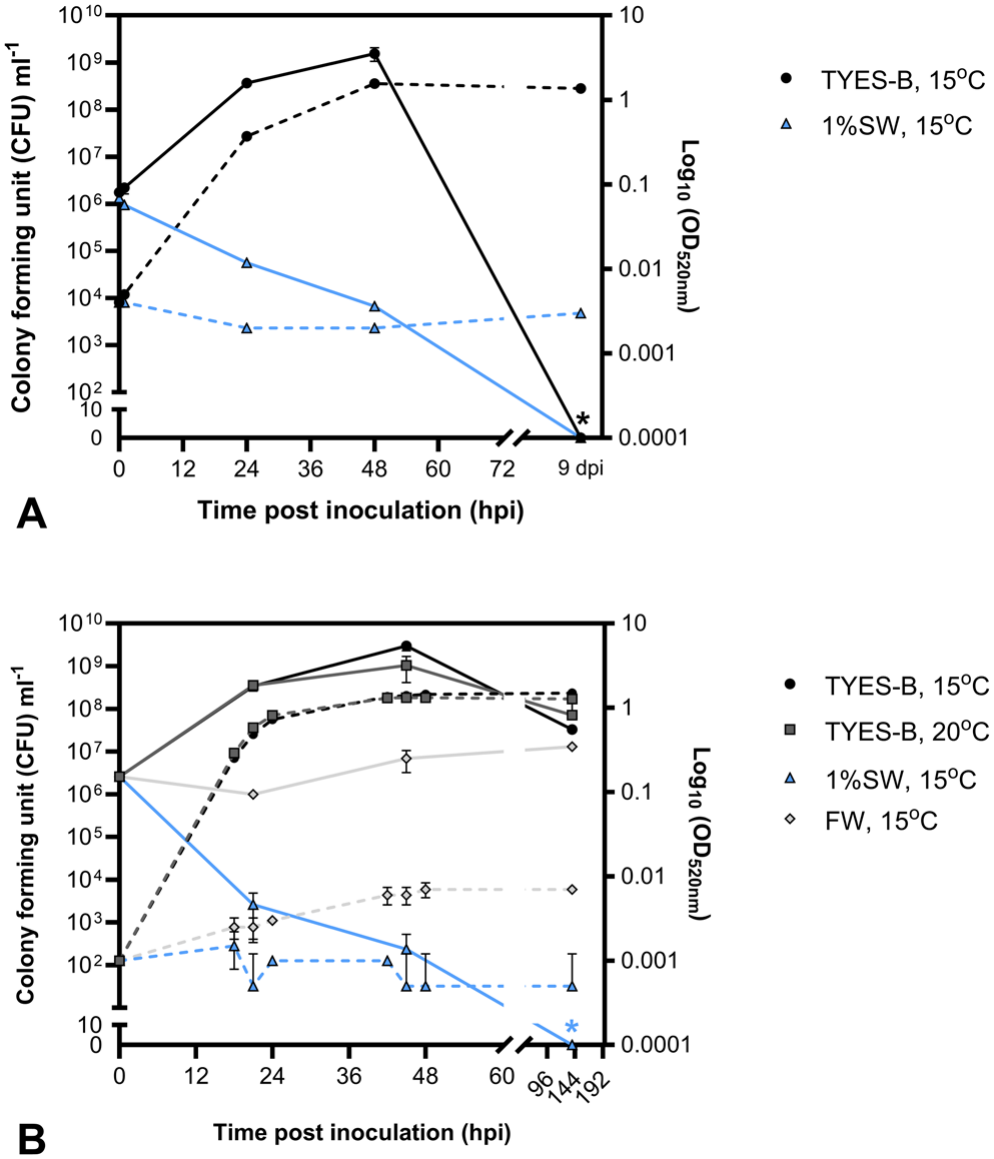
Effects of 1% water salinity (1%SW) and temperature on the growth/survival of *F. psychrophilum* 20‐1298‐14A (CFU mL^−1^: Full lines; OD_520nm_: Dotted lines). Two independent trials (A and B). (A) In CFU mL^−1^, values represent the mean and SD of two technical replicates per time point per condition. OD measurements were performed in one replicate per condition. The black asterisk indicates values below the limit of detection (TYES‐B: 1 × 10^6^ CFU mL^−1^; 1%SW: 10 CFU mL^−1^). (B) CFU and OD values represent the mean and SD of two biological replicates per time point per condition. The blue asterisk indicates values below the limit of detection for 1%SW (10 CFU mL^−1^). hpi/dpi: Hours/days post inoculation. FW: freshwater.

**FIGURE 2:**
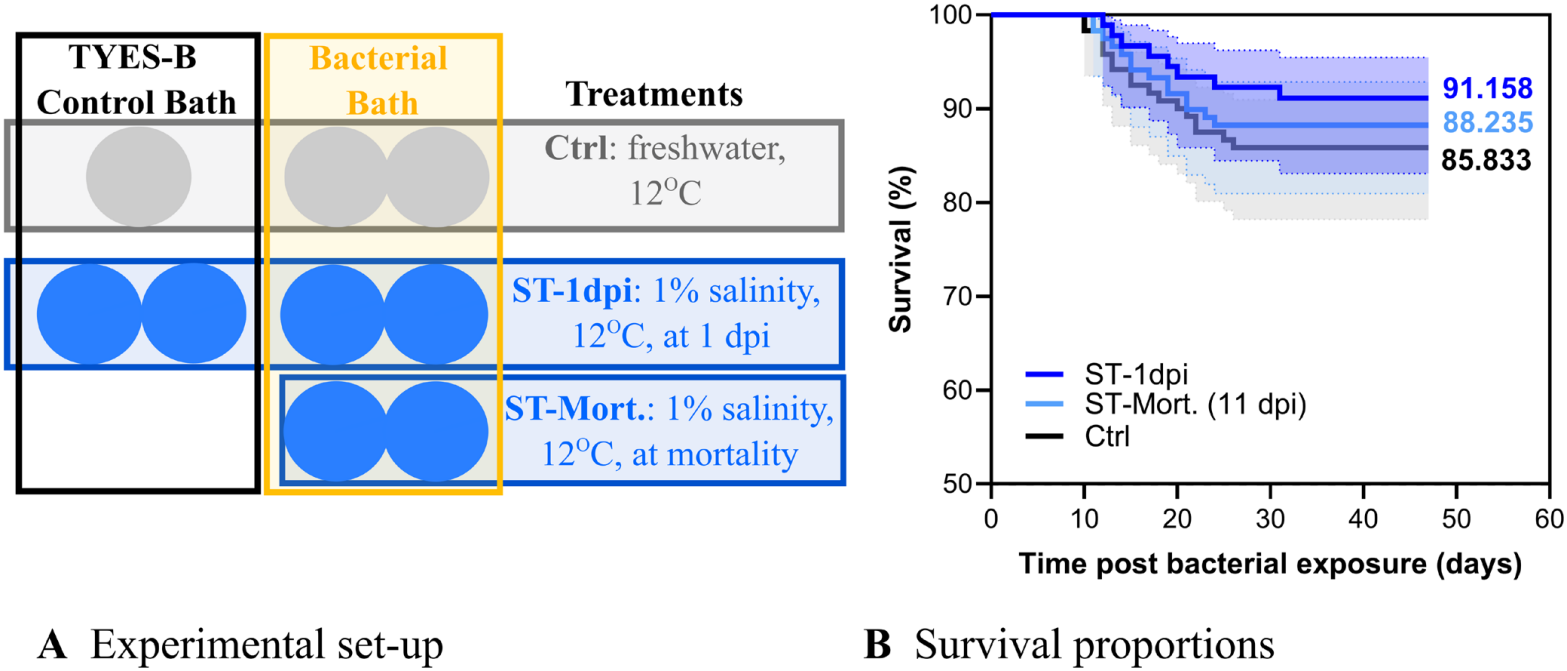
Experimental setup (A) and survival curves (B) of fish experimental challenge no. 1. (A) The circles represent the aquaria each containing 60 ± 1 rainbow trout (3–4 g). Temperature was maintained at 12°C for all aquaria while water salinity was gradually changed to 1% (10 g L^−1^) either at 1 dpi (ST‐1dpi: Salinity treatment initiated 1 dpi) or at onset of mortality (ST‐Mort. (11dpi): salinity treatment initiated at mortality, which was 11 dpi in this case). In the treatment control group (Ctrl), the water remained freshwater. The infection challenge was performed by bath (2.6 × 10^7^ CFU mL^−1^, 2 L volume, 6 h) and TYES‐B controls were included (negative controls for the infection, bath with sterile TYES‐B). (B) Mean final percent survival is indicated. 95% confidence interval (CI) is indicated. No mortality was observed for TYES‐B controls neither in freshwater (Ctrl) nor in ST (data not shown). Moribund and dead fish were positive for *F. psychrophilum* . The curves are not significantly different [Kaplan–Meier survival analysis, pairwise comparison with the Log‐rank (Mantel–Cox) test and the Gehan–Breslow–Wilcoxon test].

Table 1 gives an overview of the tested water parameters and the fish experimental infections of this study. After confirming the decreased survival of *F. psychrophilum* 20‐1298‐14A in 1%SW under laboratory conditions (Figure 1), we set up a fish infection experiment focusing on increasing the water salinity to 1% either at 1 dpi (days post infection) or at the onset of mortality (for this experiment, it was observed at 11 dpi) (Table 1 and Figure 2). The experimental set up is presented in Figure 2A (3–4 g rainbow trout, origin: Denmark). For the salinity treatment (named ST), negative controls for the infection were included only when salinity was gradually raised at 1 dpi to reduce the number of fish used. No significant effect was observed on fish survival in any of the treatment groups (Figure 2B) and final percent survival was 91.2%, 88.2% and 85.8% for fish in ST‐1 dpi, ST‐Mort. and Ctrl, respectively. All dead/moribund fish were confirmed positive to *F. psychrophilum*. At termination, the bacterium could be isolated from 1 to 3 fish per infected aquarium (always from the spleen, one fish was positive to *F. psychrophilum* in the kidney only). No mortality or signs of disease were observed in any of the three negative controls. Among the randomly chosen fish (10 per negative control aquaria) for the final sampling, there were no signs of disease in the internal organs, and no bacterial growth was observed on TYES‐A.

**FIGURE 3:**
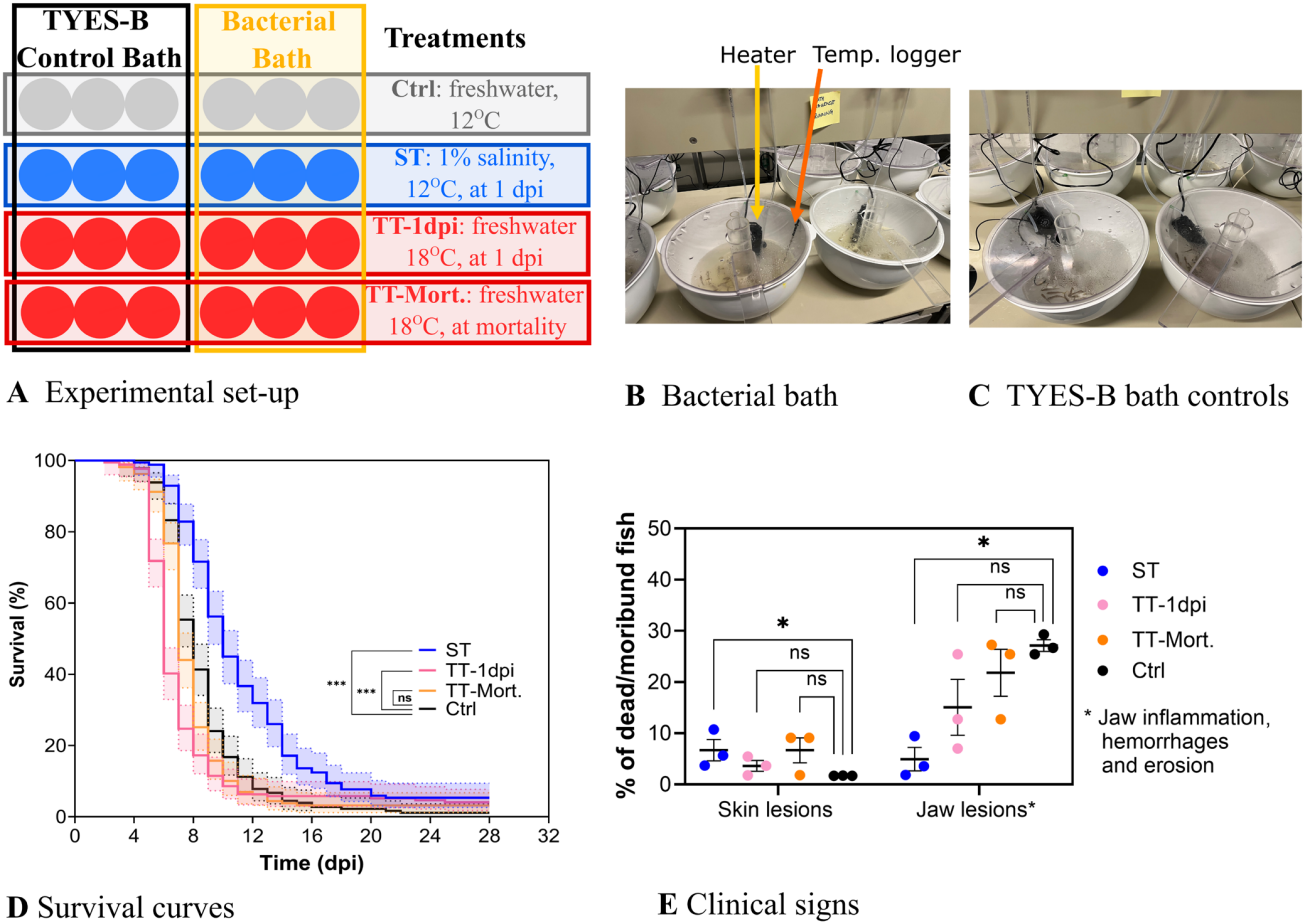
Fish experimental challenge no. 2: Effect of water salinity and temperature on RTFS. (A) Experimental set up. The circles represent the aquaria with rainbow trout fry (0.5–0.7 g). Water salinity was increased to 1% (10 g L^−1^) at 1 dpi (blue circles; ST: Salinity treatment) while temperature was increased to 18°C ± 1°C (red circles) either at 1 dpi (TT‐1dpi: Temperature treatment initiated 1 dpi) or at onset of mortality (TT‐Mort.: Temperature treatment initiated at mortality, which for this experiment was firstly observed 6 dpi). In the treatment control group (Ctrl), the water remained freshwater. In Ctrl and ST, the water temperature was maintained at 12°C. The infection challenge was performed by bath (9.0 × 10^7^ CFU mL^−1^, 2 L volume, 6 h) and TYES‐B controls (negative controls for the infection, bath with sterile TYES‐B) were included for each treatment. (B and C) Bath infection procedures, infected (B) and TYES‐B controls (C) aquaria. (D) Survival curves. Results of survival curves' comparisons with the infection control aquaria are indicated [Kaplan–Meier survival analysis, pairwise comparison with the Log‐rank (Mantel–Cox) adjusted with Bonferroni‐corrected threshold]: ***p < 0.0001. 95% confidence interval (CI) is indicated. Moribund and dead fish were positive for *F. psychrophilum* . No mortality and no signs of disease in relation to *F. psychrophilum* were observed in TYES‐B controls except two TT aquaria (see results section). (E) Percentage of skin lesions and jaw lesions (e.g., inflammation, haemorrhages and erosion) observed in dead/moribund fish following bath challenge. *p < 0.05, ns, not significant.

In a second fish experiment (Figure 3), we tested both salinity (1%) and elevated temperature (18°C ± 1°C) as possible RTFS control strategies. When tested in vitro (Figure 1), no significant effect was observed on the bacterial growth between the two selected incubation temperatures (15°C and 20°C), except for a slightly higher CFU concentration at 15°C when about to reach the stationary phase (45 hpi): 3.0 × 10^9^ ± 7.1 × 10^8^ CFU mL^−1^ at 15°C; 1.1 × 10^9^ ± 6.4 × 10^8^ CFU mL^−1^ at 20°C (mean ± SD, *n* = 2).

The experimental set up of the second challenge trial is presented in Figure 3A. As no significant difference in fish survival was observed in relation to the timing of the water salinity change in the first experiment, for this second experimental challenge water salinity was raised to 1% at 1 dpi only. For the temperature treatment (TT) groups, the water temperature was raised either at 1 dpi or at the onset of mortality (in this case at 6 dpi). Figure 3B,C illustrate the bath challenge procedure and the positioning of the heaters and temperature loggers in the aquaria.

The effects of the tested treatments on fish survival are presented in Figure 3D. Even if no significant differences in the final percent survival were observed, survival curves of fish exposed to ST and the ones exposed to TT‐1dpi were significantly different from the survival curve of the infected control (*p* value < 0.0001). While ST caused a delay in the development of disease compared to the un‐treated control (Ctrl), the elevated temperature (TT aquaria) accelerated the disease/mortality compared to the un‐treated control (Ctrl). As an example, 20% fish survival was reached at 14 dpi in ST, at 7–8 dpi in TT‐1dpi, at 8–9 dpi in TT‐Mort. and at 9–10 dpi in Ctrl. Similarly, fish reached 10% survival between 17 and 21 dpi in ST tanks, at 9–10 dpi in TT‐1dpi, at 10–11 dpi in TT‐Mort. and at 11–12 dpi in Ctrl. Among the clinical signs characterising dead/moribund fish (Figure 3E), we observed skin lesions and jaw lesions such as haemorrhages and erosions. A significant increase in skin lesions (6.7% in ST, 1.7% in Ctrl; adjusted *p* = 0.04) and a significant decrease in jaw lesions (4.9% in ST, 27.1% in Ctrl; adjusted *p* = 0.008) were observed in dead/moribund fish in ST compared to Ctrl.

Among the TYES‐B controls in the TT groups, we observed signs of disease such as dark coloration, swollen abdomen and erosion of the jaw and mortalities due to *F. psychrophilum* in two aquaria (one per temperature treatment). The cause of mortality (between 80% and 40% between 9 and 25 dpi) was confirmed by the re‐isolation of the bacterium, which was further characterised, e.g., MALDI‐TOF MS and serotyping, to confirm the contamination. This contamination was attributed to the accidental transfer of *F. psychrophilum* from neighbouring infected aquaria, possibly during the daily cleaning procedures. The contamination was limited to these two aquaria as no mortality and no signs of disease in relation to *F. psychrophilum* were observed in the other negative control aquaria. In addition, it was also possible to re‐isolate the bacterium from randomly chosen fish at the end of the experiment for these two aquaria. In the other TYES‐B control groups instead, there were no visually relevant signs of disease in fish at termination as well as no signs of disease in the internal organs and no bacterial growth on TYES‐A among the randomly chosen 10 fish for the final sampling. Among the remaining fish in the bacterial exposed aquaria, it was possible to re‐isolate the bacterium from the brain of 3 out of 11 fish in ST triplicate aquaria, from the spleen of one fish out of 7 in TT‐1 dpi triplicate aquaria, and none of the 3 survived fish in the TT‐Mort. group.

To further evaluate the prophylactic effect of ST on RTFS, TYES‐B control fish kept in freshwater (Ctrl) or ST during the second fish experiment were used for a co‐habitation challenge experiment (fish experiment no. 3). No *F. psychrophilum* infection was detected—no observation of clinical signs and no bacterial growth detected after streaking of internal organs on TYES‐A or blood‐agar—in the few individuals dying in these groups (1–2 fish per aquarium) during the second experiment, and the fish were thus considered to be *F. psychrophilum* ‐free.

**FIGURE 4:**
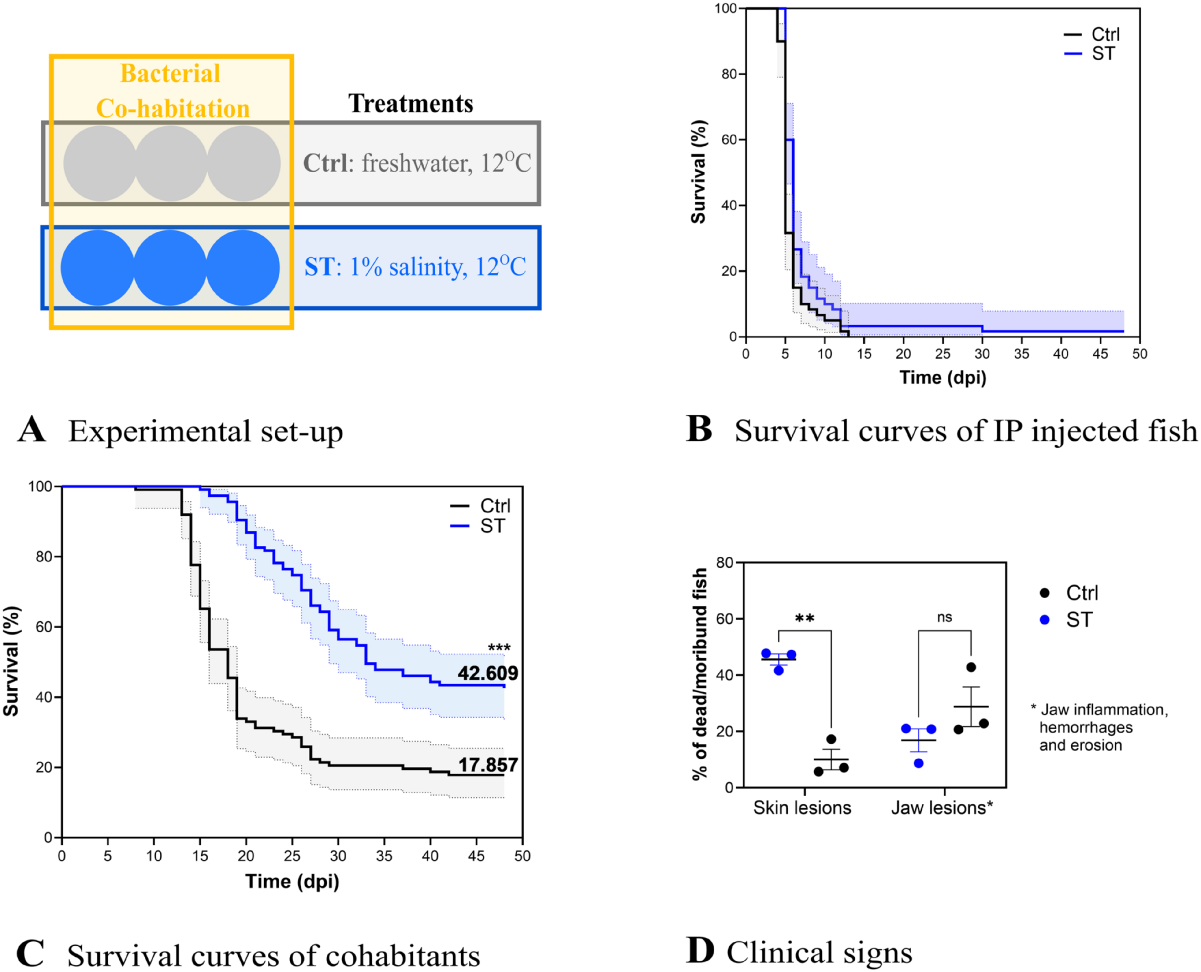
Fish experimental challenge no. 3: Co‐habitation challenge and effect of water salinity. (A) Experimental set up. The circles represent the aquaria containing each 59 ± 2 rainbow trout fry (1–2 g). Water salinity was changed gradually to 1% (10 g L^−1^) 30 days before the infection (ST: Salinity treatment). In the treatment control group (Ctrl), the water remained freshwater. Temperature was maintained at 12°C for all aquaria. The infection challenge was performed by co‐habitation, where 20 fish in each aquarium were IP injected (50 μL, 5.5 × 10^3^ CFU fish^−1^) and fin clipped (dorsal caudal fin) to allow recognition. TYES‐B control aquaria were not included for this experiment (see materials and methods). (B) Survival over time of fish injected by IP with the bacteria. Moribund and dead fish were positive for *F. psychrophilum* . (C) Survival over time in the experimental groups exposed to *F. psychrophilum* by cohabitation (cohabitants). Final percentages and 95% confident interval (CI) are shown for each curve. The curves are significantly different [Kaplan–Meier survival analysis, pairwise comparison with the Log‐rank (Mantel–Cox) test and the Gehan‐Breslow‐Wilcoxon test], ***p < 0.0001. Moribund and dead fish were positive *F. psychrophilum* . (D) Percentage of skin lesions and jaw haemorrhages/erosion observed in dead/moribund fish in ST (*n* = 3) and positive infection control aquaria (*n* = 3) during cohabitation challenge. Mean and SD are indicated. **p = 0.001, ns, not significant and *p* value > 0.05.

IP injected seeder fish reached 0% survival within 2 weeks in both groups (Figure 4B). Cohabitant fish in ST had a significantly higher survival (42.6%) compared to cohabitant fish in Ctrl (17.9%) (*p* value < 0.0001) (Figure 4C). The replicate aquaria were characterised by homogenous survival rates among them (Ctrl‐IPseeders: 0, 0, 0%; Ctrl‐cohabitants: 10, 24, 20%; ST‐IPseeders: 5, 0, 0%; ST‐cohabitants: 33, 51, 43%). Infected dead and moribund fish were confirmed positive for *F. psychrophilum*. Among the clinical signs characterising dead/moribund fish in ST and control aquaria (infected groups) (Figure 4D), we observed skin lesions (45.62% in ST, 10.03% in Ctrl; *p* = 0.001) and jaw haemorrhages/erosions (16.86% in ST, 28.80% in Ctrl; not significant). All the fish surviving the infection were inspected for *F. psychrophilum*. The bacterium was recovered from 1 out of 12 (only kidney), 1 out of 17 (kidney and brain) and 1 out of 20 (only kidney) survivors in the triplicate aquaria in ST and from 1 out of 4 (only kidney), 1 out of 7 (only brain) and 0 out of 9 survivors in the Ctrl aquaria.

## 4 Discussion

Environmental factors play a crucial role in the development of infectious diseases. In our study, we wanted to evaluate the impact of altering water salinity and water temperature, as possible practical strategies to counteract *Flavobacterium psychrophilum* infection development and spreading in aquaculture facilities.

Salt is often used as a stress mitigator during fish transport. The addition of 5 g L^−1^ of NaCl (0.5%) to transport water was shown to ameliorate stress parameters (e.g., enhanced tight junction and mucin gene expression) and reduce skin bacteria growth in rainbow trout (200 g) (Tacchi et al. 2015). In the case of *Yersinia ruckeri* , it was shown that increased salinities from 1‰ to 9‰ caused a reduction in rainbow trout mortality (Altinok and Grizzle 2001).

*Flavobacterium psychrophilum* isolates can tolerate up to 1% NaCl in laboratory conditions (Bernardet and Kerouault 1989; Dalsgaard and Madsen 2000; Madetoja et al. 2001), and no report has shown the ability of any *F. psychrophilum* isolate to grow in higher salt concentrations (Madetoja et al. 2003). Previous in vitro studies have suggested the possibility of using salinity at or above 10 g L^−1^ (1%) as a controlling agent for this bacterial infection (Soltani and Burke 1994). In our study, we assessed the efficacy of increasing the salinity of the water to 1% on enhancing fish survival after *F. psychrophilum* exposure. In the experiments presented, the addition of sodium chloride to the water caused a delay in the development of disease when added therapeutically 1 day post bacterial bath exposure (fish experiment no. 2) and showed a significant effect in reducing cohabitant‐fish mortality when the salinity treatment was used as a prophylaxis (fish experiment no. 3). Taking the reduced survival of the bacteria in ST compared to Ctrl (freshwater), these results suggest that the ST decreased the bacterial exposure of the co‐habitants and hereby reduced the spread of infection between fish. Further studies are needed to pinpoint whether ST also leads to reduced release of bacteria from infected fish, and/or reduced infectivity/entry. The role of fish skin and its microbiota should also be investigated in this specific infection as it may play a critical role as demonstrated in the case of external stress factors, for example, stress (Tacchi et al. 2015). In terms of clinical symptoms, increased salinity appeared to reduce jaw pathology but to promote skin lesions among fish succumbing following bacterial exposure. Detailed studies of infection routes and spread/replication of *F. psychrophilum* inside the fish are needed to determine whether/how the saline condition affects propagation of disease/pathology. Altogether, the obtained results in the fish experimental trials suggest that increasing the water salinity to 1% is a promising treatment for delaying and partly reducing losses caused by RTFS.

In contrast to what was observed for water salinity, the selected increase of water temperature did not have a beneficial effect on fish survival and infection development. While *F. psychrophilum* grows at temperatures between 4°C and 20°C in vitro (some strains up to 25°C; 15°C is considered its optimal in vitro growth temperature) (Bernardet and Kerouault 1989; Dalsgaard and Madsen 2000; Madetoja et al. 2001; Sugahara, Fujiwara‐Nagata, and Eguchi 2010; Sugahara, Fujiwara‐Nagata, Fukuda, and Eguchi 2010), the disease in rainbow trout in the field generally manifests when water temperature ranges between 3°C and 15°C (Madetoja and Wiklund 2002). Previous in vitro studies have suggested the possibility of using temperatures above 20°C as a controlling agent for this bacterial infection (Soltani and Burke 1994). However, fish are poikilotherm animals and, rainbow trout is a cold‐water fish that do not tolerate temperatures outside of their specific range: 9°C–15°C optimal range with an optimal temperature of 11°C–12°C; possible growth between 5°C and 18°C; survival up to 24°C; lethal above 25°C (Woynarovich et al. 2011). Thus, in our experiment, we decided to raise the water temperature to 18°C ± 1°C and not above 20°C.

At a first glance, our results are in contrast with the results of previous studies reporting a beneficial effect of high temperature, as in our experiment the increased temperature speeded up the infection (Lorenzen and Olesen 1996b; Jensen et al. 2003). However, differences in the applied temperatures (18°C vs. 21°C) as well as bacterial and fish inter‐strain diversity might explain these divergent observations. Lorenzen and Olesen (1996b) observed a positive effect on *F. psychrophilum*‐challenged rainbow trout fry (1 g) survival at a temperature of 21°C compared to when at 10°C and 15°C (the water temperature was changed 24 h after infection procedures). The authors suggested that the immune system of the fry could have been stimulated by the increased water temperature. Similarly, a significant increase in survival was observed in *F. psychrophilum*‐challenged rainbow trout juveniles (3.5 g) at a temperature of 18°C and 23°C (fish were tempered to water temperature prior to infection) compared to the tested low temperatures (77%–96% mortality at temperatures between 3°C and 15°C; 38.0% mortality at 18°C, 8% at 21°C and 0% at 23°C) (Holt et al. 1989). The authors concluded that by increasing the water temperature to 18°C–23°C the advantage shifts to the host as the host response is more rapid and the bacterial growth slower than in lower temperatures (below 15°C). In addition, elevating the water temperature to 22°C seemed to lower the mortality of rainbow trout fry (2 g) in characterisation experiments of a recently identified *F. psychrophilum* isolate in South Korea (injected by IP and intramuscularly (IM)). However, the experiments were run with a small number of fish (7–10 fish per 10 L aquaria) and without any replicate aquaria per condition (Park et al. 2023). In other fish species e.g., ayu ( *Plecoglossus altivelis* ), the warmer water temperature (22°C) has however shown a decreased resistance to this bacterial infection and an increased inflammatory response (0.5 g fish) (Kato et al. 2023) where 28°C (for three consecutive days) has instead shown an increased resistance to this infection in ~4 g fish (Sugahara, Fujiwara‐Nagata, and Eguchi 2010; Sugahara and Eguchi 2012).

Although not significant, the disease‐promoting effect of increased water temperature was higher when initiated at 1 dpi compared to 6 dpi in our experiment. This suggests that the elevated temperature was favouring the bacterium in the host‐pathogen battle. Importantly, comparison of different studies dealing with temperature effects on host‐pathogen interactions in fish should take both pathogen isolate and fish strain/species into account. Indeed, *F. psychrophilum* strain diversity (Sundell et al. 2019; Bruce et al. 2021) as well as rainbow trout diversity (Marancik et al. 2014; Wiens et al. 2018; Mathiessen et al. 2023) have a large impact in the development and severity of RTFS. Thus, to further investigate the effect of increased water temperature within this context, fish experiments should be repeated with larger inter‐strain diversity of *F. psychrophilum*, for example, isolates with a lower virulence, as well as rainbow trout of a different origin. These experiments would bring more knowledge and understanding not only on how and if increasing water temperature would reduce RTFS development, but also on how warmer water temperatures in relation to climate change might impact the development and severity of fish diseases in aquaculture facilities, as for example it may be for piscirickettsiosis in Atlantic salmon (Bravo et al. 2020).

The overall aim of the study was to create practical strategies against RTFS that can easily be used in the field. Thus, a collaboration with a Danish recirculation aquaculture system (RAS) fish farm (rainbow trout fry farm with RTFS outbreaks) was established and a preliminary field trial, based on the results obtained in the infection facilities, was performed (NaCl was added to one of the two strings with rainbow trout fry). Even though the overall mortality was similar in both groups (14%–20%), the infection was 1 week delayed in the group with increased water salinity (0.5%) supporting the findings of the laboratory experiments (Schmidt et al. 2025). The salt concentration was lowered compared to the lab experiments, due to farm management operational capacity.

## 5 Conclusion and Future Perspectives

Finding alternative methods to antibiotics for the control and spread of bacterial diseases in aquaculture facilities is of great importance for reducing the spread of antimicrobial resistance in aquatic pathogens. With this study, we assessed the possible use of altering environmental parameters as a control method of *Flavobacterium psychrophilum* infections. Increasing water salinity proved to slow down disease progression and to partly reduce mortality associated with experimentally induced RTFS. In applied terms, the delayed progression may give time to isolate and grow the causing *F. psychrophilum* , characterise its antimicrobial resistance profile to evaluate antimicrobials efficacy, and so initiate treatment before reaching heavy mortalities. This would support prudent use of antimicrobials. In future projects, it would be valuable to evaluate the robustness of the preventive effect of this treatment and its effect on the microbial communities (fish and farm environment). Further, it would be valuable to investigate if and how increasing water salinity could be used in synergy with other preventive approaches, for example, phage therapy.

## Author Contributions

**Valentina L. Donati:** conceptualization, investigation, methodology, validation, data curation, visualization, formal analysis, writing – original draft and review and editing. **Niels Lorenzen:** funding acquisition, project administration, writing – review and editing. **Lone Madsen:** conceptualization, investigation, methodology, funding acquisition, project administration, writing – review and editing, supervision.

## Conflicts of Interest

The authors declare no conflicts of interest.

## Data Availability

The original contributions presented are included in the article. Further inquiries can be directed to the corresponding author.
